# Post-exercise effects of self-selected exercise intensity on cardiovascular parameters in peripheral artery disease: a randomized crossover study

**DOI:** 10.31744/einstein_journal/2025AO1498

**Published:** 2025-08-25

**Authors:** Max Duarte de Oliveira, Hélcio Kanegusuku, Deivide Rafael Gomes de Faria, Tiago de Oliveira Peçanha, Nelson Wolosker, Marilia de Almeida Correia, Raphael Mendes Ritti-Dias

**Affiliations:** 1 Universidade Nove de Julio São Paulo SP Brazil Postgraduate Program in Rehabilitation Science, Universidade Nove de Julio, São Paulo, SP, Brazil.; 2 Hospital Israelita Albert Einstein São Paulo SP Brazil Hospital Israelita Albert Einstein São Paulo, SP, Brazil.; 3 Manchester Metropolitan University Institute of Sport Department of Sport and Exercise Sciences Manchester UK Department of Sport and Exercise Sciences, Manchester Metropolitan University Institute of Sport, Manchester, UK.

**Keywords:** Intermittent claudication, Peripheral arterial disease, Vascular diseases, Blood pressure, Heart rate, Walking

## Abstract

**Objective:**

To analyze the post-exercise effects of self-selected exercise intensity compared with the intensity recommended by guidelines on blood pressure and heart rate variability in participants with peripheral artery disease.

**Methods:**

This was a randomized crossover trial. Twenty participants with peripheral artery disease (52.6% men; 69.1±7.9 years; ankle-brachial index 0.59±0.15) underwent three experimental sessions: self-selected exercise intensity, intensity recommended by guidelines, and a control session (no exercise). Blood pressure (auscultatory method), heart rate, and heart rate variability were measured before and after the intervention.

**Results:**

Acute reductions in systolic blood pressure were observed in the exercise sessions compared to the control (p<0.005). The heart rate decreased after control and increased after exercise sessions (p<0.001). The absolute heart rate increase after exercise sessions were higher than that in the Control Group p<0.001. Heart rate recovery showed a progressive decrease post-session over 300 s (p<0.001), with the root mean square of successive differences significantly decreasing up to 60 seconds in both exercise sessions (p<0.05).

**Conclusion:**

Acute walking exercise sessions prescribed at self-selected exercise intensity had similar effects on reducing systolic blood pressure and heart rate recovery compared with walking exercise session performed at the guideline-recommended intensity.

**ClinicalTrials.gov Identifier:**

NCT04333615.

## In Brief

This randomized crossover trial evaluated the acute effects of a self-selected intensity walking session compared to the intensity recommended by guidelines on cardiovascular outcomes in patients with peripheral artery disease. These findings demonstrate that walking at a self-paced intensity provides comparable reductions in systolic blood pressure and similar patterns of heart rate recovery as the intensity recommended by the guidelines. These results highlight the potential of self-selected-intensity exercise as a feasible and effective strategy for clinical exercise prescription in patients with peripheral artery disease.

## Highlights

■ A single session of self-selected intensity walking reduced systolic blood pressure in individuals with peripheral artery disease.
■ Heart rate recovery responses after self-selected-intensity walking were similar to those following the guideline-prescribed exercise.
■ Self-selected intensity exercise may offer a feasible strategy for patients with claudication, balancing comfort and cardiovascular benefits.
■ These findings support flexible exercise prescriptions tailored to individual comfort without compromising the cardiovascular benefits.

## Graphical abstract

**Figure:**
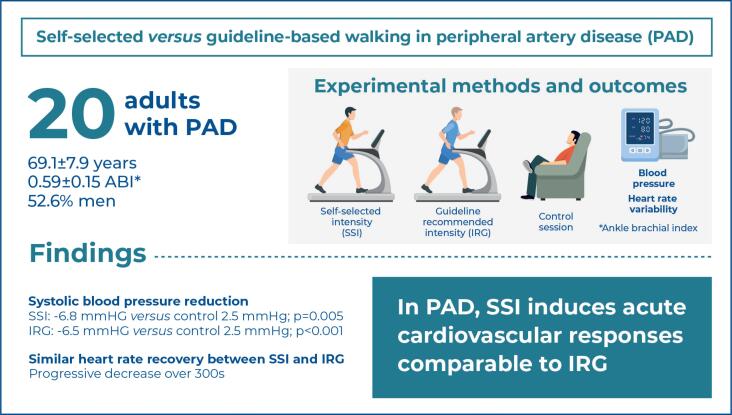

## INTRODUCTION

Peripheral artery disease (PAD) is a chronic vascular disorder characterized by constriction of arteries in peripheral regions of the body.^(1)^ Intermittent claudication, the most common symptom of PAD, prevents patients from engaging in routine physical tasks, leading to diminished levels of physical activity.^(2)^ In addition to walking impairment, these patients have a high risk for cardiovascular diseases.^(3)^ Hypertension is common in this population and has been associated with greater arterial stiffness, regardless of other clinical factors. Uncontrolled hypertension is more frequent in older patients, those with a lower ankle-brachial index, and those who do not use ACE inhibitors.^(4)^ Therefore, interventions to reduce blood pressure have been recommended.^(5)^

In patients with claudication, a session of walking exercise performed at the onset of pain, as prescribed by established guidelines,^(1)^ leads to a reduction in post-exercise blood pressure.^(6)^ However, claudication-induced pain has been described as the primary barrier to the engagement of these patients in physical activities.^(7)^ A potential strategy to overcome this barrier is prescribing a self-selected exercise intensity, thereby enabling individuals to choose their exercise intensity.^(8,9)^ This strategy has been associated with increased pleasure during exercise in several populations, suggesting a broader understanding of pleasure beyond conventional definitions such as satisfaction, gratification, and positive emotional experiences in the context of exercise.^(8)^ Patients with PAD performed walking exercises at a slightly lower intensity with longer bout durations than the intensity recommended by the guidelines.^(10)^ Notably, cardiovascular stress and total distance covered during exercise were similar between the sessions. However, whether these cardiovascular responses during exercise translate into similar post-exercise effects, remains unclear.

Acute reductions in blood pressure after a single exercise session have been considered clinically relevant, as blood pressure reductions occur with sufficient magnitude and duration and can stay lowered for several hours.^(11)^ In patients with PAD, acute reductions in blood pressure have been observed after walking,^(12)^ resistance training,^(13,14)^ arm cranking^(15)^ and Tai Chi Chuan^(16)^ exercises, indicating that these patients can also experience acute cardiovascular benefits with exercise. Beyond blood pressure responses, assessing cardiac autonomic modulation recovery after exercise allows us to understand the transient cardiovascular adjustments of the parasympathetic and sympathetic systems, which are directly related to the integrity of cardiovascular autonomic control.^(17)^ The cardiac autonomic recovery stabilizes within 300 s post-exercise, reflecting the reestablishment of autonomic balance.^(17)^ Furthermore, exercise training improves heart rate recovery, promotes parasympathetic reactivation, and reduces sympathetic activity in patients with cardiovascular impairment.^(18)^ Late recovery of the heart rate has been associated with increased cardiovascular risk and mortality, highlighting its clinical relevance in populations with autonomic dysfunction.^(19)^ Furthermore, exercise training improves heart rate recovery by promoting parasympathetic reactivation and reducing sympathetic activity in patients with cardiovascular impairment.^(19)^ Nevertheless, it is unknown whether self-selected-intensity walking exercises can yield benefits, specifically post-exercise blood pressure reductions and heart rate recovery, compared to guideline-recommended intensities. Understanting the physiological responses of self-selected exercise in patients with PAD could support more personalized and sustainable exercise prescriptions.

## OBJECTIVE

This study aimed to analyze the acute post-exercise effects of a walking session prescribed with a self-selected intensity on cardiovascular parameters in patients with peripheral artery disease, comparing it to the current intensity recommended by the guidelines.

## METHODS

### Study design, setting, and ethics

This was a phase III, single-blind, randomized crossover clinical trial designed to minimize inter-individual variability by allowing each participant to serve as their own control. A crossover design was chosen because the within-patient variation was lower than the between-patient variation, improving the statistical power with a smaller sample size. Given the acute nature of the interventions, a 48-hour washout period was implemented to eliminate potential carryover effects, as hemodynamic and cardiovascular responses typically return to baseline within this timeframe. Patients with PAD and claudication symptoms were recruited from hospitals in São Paulo City between September 2021 and July 2022 and randomized in a 1:1 ratio, ensuring that each participant started with either a self-selected intensity session or a guideline-recommended session in a counterbalanced order.

This study was approved by the Research Ethics Committee of the *Universidade Nove de Julio*, CAAE: 19372819.6.0000.5511; #4.944.107. The patients were informed about all procedures and the potential risks and benefits of the study, and those who agreed to participate signed a written informed consent form prior to participation. This study adhered to the recommendations of the Consolidated Standards for Reporting Trials. The study data were collected and managed using the REDCap electronic data capture tools hosted at *Universidade Nove de Julho*.

### Participants

Patients were included in the study if they: I) were aged ≥50 years; II) presented an ankle-brachial index (ABI) <0.90 in one or both limbs;^(20)^ III) presented claudication symptoms during the six-minute walking test;^(21)^ IV) were able to walk at least 90 m without interruption; and VI) had no amputated limbs and/or ulcers.^(21,22)^

### Randomization and allocation

Simple randomization was performed in this study. A researcher not directly involved in recruitment or data collection generated the allocation sequence using an open web-based system (https://www.randomizer.org) to generate the code. In addition, the allocation information was concealed from the researchers who performed the measurements.

### Protocol

Patients performed three experimental sessions in random order: walking exercise with intensity recommended as per guidelines (30 min of exercise split into sets of 3-5 minutes at an intensity that elicits moderate to maximum pain);^(1)^ walking exercise with self-selected intensity (30 min of exercise with patients choosing the speed and duration of sets, regardless of the occurrence of pain), and control (30 min resting on a treadmill).

The sessions were performed at least 3 days apart, at the same time of day, between 8:00 AM and 5:00 PM. Patients were instructed to abstain from any vigorous physical activity the day before the session, eat a light meal 2 h before the session, avoid caffeinated or alcoholic beverages and refrain from smoking on the test days, and to maintain normal medication use.

During each experimental session, a 10-minute rest period in the supine position was followed by the measurement of heart rate and brachial blood pressure, both measured in triplicate. Subsequently, the R-R intervals were recorded for 10 min to evaluate the heart rate variability. Heart rate recovery was assessed immediately after the intervention by analyzing R-R interval recordings while standing on the treadmill. Subsequently, the patients returned to the supine position for a 30-minute period, during which all previously conducted procedures were repeated. Sensitivity measures were considered by standardizing the data collection conditions and controlling for external factors such as prior physical activity, diet, and medication use. Reproducibility was ensured through triplicate measurements, and validated protocols were used for all the assessments. Details of the assessment of patient characteristics are described in Tables 1S-3S, Supplemental Materials).

### Primary outcomes

The systolic and diastolic brachial blood pressures were the primary outcomes measured using the auscultatory method with a mercury sphygmomanometer. Three consecutive measurements were performed at 1-minute intervals using an appropriate cuff size for the arm circumference. The value used was the average of the three measurements.

### Secondary outcomes

Cardiac autonomic modulation was assessed using heart rate variability and heart rate recovery analyses. The heart rate variability and heart rate recovery were evaluated from the R-R intervals obtained using a heart rate transmitter (Polar H10, Polar, Finland), in which the signals were sent synchronously to a heart rate monitor valid for this function (V800, Polar^®^, Finland). For baseline cardiac autonomic modulation, stationary data were recorded for 10 min with the patient in the supine position, following a previously described protocol,^(22)^ with at least 5 min used for analysis. All stationary analyzes were performed using the Kubios HRV Standard software (version 3.4.3, Biosignal Analysis and Medical Imaging Group, Finland), following the recommendations of the Task Force for heart rate variability.^(23)^

The analysis of heart rate recovery and post-exercise heart rate variability was based on the signals recorded during the last 30s of the intervention and 5 min after the end of the intervention using the SinusCor program (version 1.0.2) operated in MATLAB (MathWorks, Massachusetts, USA).^(24)^ Through this program, it was possible to perform a time-varying analysis using the windowing function with segments of 30s and the quotient filter and subsequent calculation of the indices: standard deviation of all normal-to-normal intervals (SDNN) and root mean square differences between adjacent normal RR intervals (RMSSD) at 30s intervals.^(24)^

The following time-domain parameters were obtained for the analysis of stationary and/or non-stationary data: SDNN, RMSSD, and pNN50. SDNN was accepted as a marker of total variability (sympathetic and parasympathetic cardiac modulations) and RMSSD and pNN50 were accepted as markers of predominant cardiac parasympathetic modulation.^(23)^ Frequency domain variables were calculated using Fast Fourier Transform. Spectral components were assigned based on their center frequency as low (0.04-0.15 Hz) and high (0.15-0.4 Hz) frequencies. The low- and high-frequency components were accepted as markers of predominant sympathetic and parasympathetic cardiac modulations, respectively, and the low-frequency/high-frequency ratio was considered the cardiac sympathovagal balance.^(23)^

### Statistical analysis

The sample size was calculated using G*Power (version 3.1.9.7, Germany), considering a power of 80% (β=0.20), an alpha error of 0.05, and a medium effect size (d=0.5). As no similar studies were found in the literature, the effect size was estimated arbitrarily based on the standard conventions for detecting moderate effects on physiological responses. The final estimate indicated a sample size of 13 patients (39 sessions).

The normality of the data distribution and homogeneity of variance were confirmed using the Shapiro-Wilk test. Continuous variables were described as mean and standard deviation, and categorical variables were described as relative frequencies. For each evaluated session, the values of Δ (difference between two measurements) were determined, based on the following formula: Δ = post 30 minutes-pre moment. To compare the baseline cardiovascular parameters and Δ, a one-way ANOVA with post-hoc Bonferroni test was used. To analyze the acute effect, Generalized Estimation Equations of two factors (session and time) using unstructured correlation, a robust covariance matrix, and gamma distribution were used. Post-hoc comparisons were performed using Bonferroni's complementary test to identify differences. Statistical analyses were performed using SPSS (version 25.0; SPSS Inc., Chicago, IL, USA). P was set at p<0.05. significant.

## RESULTS

### Population and characteristics

**Figure 1:**
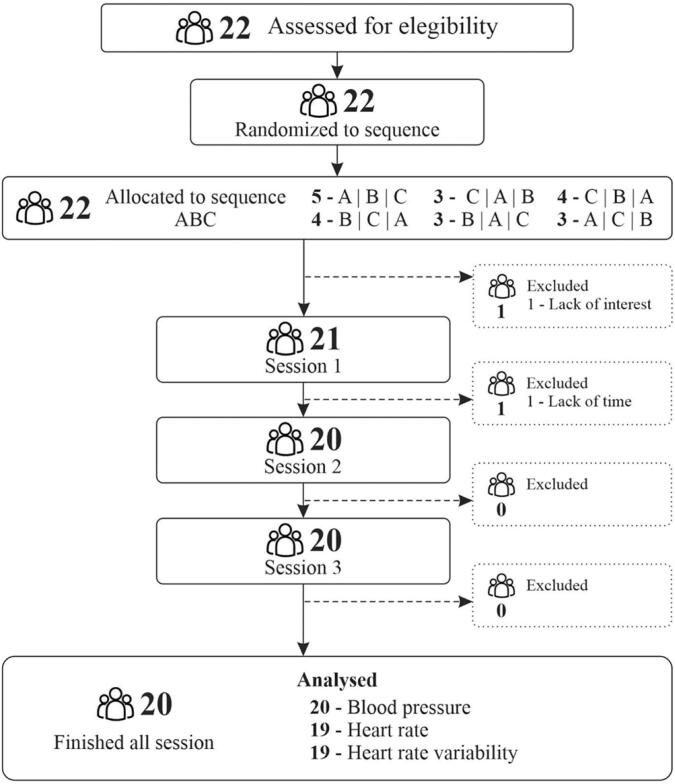
Recruitment of participants process

A flowchart of the participant recruitment process is shown in figure 1. Twenty-two individuals expressed interest in participating in the study, of which 21 met the inclusion criteria and actively participated. One patient dropped out of the study along the experimental sessions. Twenty patients completed all three experimental sessions. Data on heart rate and heart rate variability were missing for one patient owing to equipment problems. Most participants were male, with risk factors such as dyslipidemia (73.6%), hypertension (63.2%), and diabetes (42.1%). The most frequently prescribed drugs were statins (83.3%) and antiplatelet agents (77.6%). The characteristics of these samples are listed in table 1.

A: intensity recommended by the guidelines; B: self-selected intensity; C: control.

**Table 1:** General characteristics of participants

Variables		Values (n=20)
Clinical characteristics		
	Age (years)	69.1±7.9
	Sex (%, men)	52.6
	Body mass Index (kg/m²)	27.2±5.7
	Ankle-brachial Index	0.59±0.15
Risk factors, %		
	Coronary artery disease	31.5
	History of COVID-19	20.0
	Diabetes	42.1
	Dyslipidemia	73.6
	Hypertension	63.2
	Obesity	52.6
	Renal failure	5.2
	Current smoker	26.3
	Stroke	31.5
Medication, %		
	Angiotensin converting enzyme inhibitors	27.7
	Angiotensin II receptor antagonists	38.8
	Antiplatelet	77.6
	Beta blockers	33.3
	Calcium blocker	27.7
	Diuretics	27.7
	Peripheral vasodilator	22.2
	Statin	83.3

Data are presented as mean±standard deviation or as relative frequencies.

### Primary outcomes

The baseline cardiovascular parameters during the sessions are presented in table 2. Blood pressure and heart rate were similar between the sessions (p=0.128).

**Table 2:** Cardiovascular parameters at baseline in the three sessions

	Intensity recommended by the guidelines	Self-selected intensity	Control	p value
Systolic blood pressure, mmHg	123 (117; 129)	119 (114; 124)	121 (115; 128)	0.635
Diastolic blood pressure, mmHg	69 (65; 73)	69 (64; 74)	70(66; 73)	0.955
Heart rate, bpm*	71 (64; 77)	69 (62; 75)	71 (63; 77)	0.927
RMSSD, ms*	33.4 (26.1; 42.7)	39.1 (28.3;52.2)	44.5 (33.4; 59.0)	0.143
SDNN, ms*	30.0 (20.9; 40.7)	29.9 (22.7; 40.7)	36.3 (26.3; 48.8)	0.128
Pnn50, %*	22.0 (10.6; 35.9)	33.0 (20.6; 48.9)	28.0 (17.5; 41.5)	0.408
LF, n.u*	44.2 (31.6; 49.1)	32.5 (27.6; 43.9)	25.9 (25.7; 42.8)	0.311
HF, n.u*	54.9 (50.6; 57.9)	66.2 (56.3; 73.6)	74.1 (61.4; 76.9)	0.179
LF/HF*	3.0 (0.5; 3.1)	2.6 (-1.3; 9.4)	1.1 (-1.5; 6.7)	0.249

Data are presented as mean and 95% confidence interval (95%CI).

* (n=19).

RMSSD: root mean square of successive differences; SDNN: standard deviation of all normal-to-normal intervals; Pnn50: percentage of adjacent NN intervals that differ from each other by more than 50ms; LF: low-frequency; HF: high-frequency.

**Figure 2:**
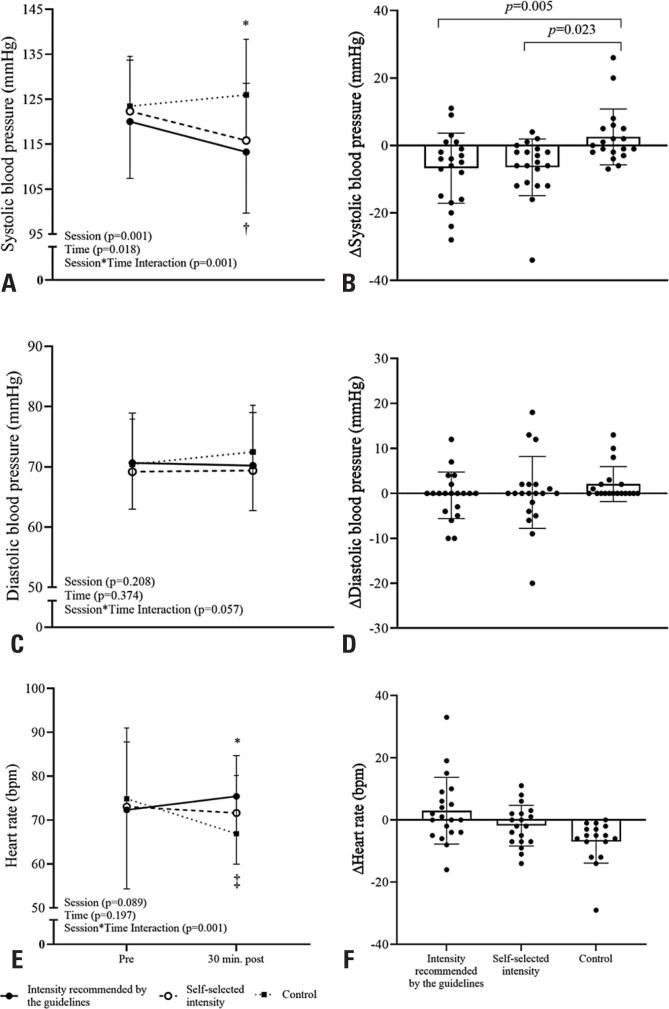
Response of cardiovascular parameters between sessions after intervention and delta systolic blood pressure in patients with peripheral arterial disease

Figure 2A shows the responses of the cardiovascular parameters pre-and 30 min post-intervention. Compared to pre-intervention, the systolic blood pressure decreased similarly in exercise sessions. Post-intervention systolic blood pressure was significantly lower during the exercise sessions than during the control sessions (p<0.05). Absolute reductions (Figure 2B) in systolic blood pressure during the exercise sessions were higher than those during the control session (p<0.05). The diastolic blood pressure did not change after the interventions (p>0.05) (Figures 2 C and D).

* Significant difference between the exercise sessions and the control; ^†^ Significant difference of the self-selected intensity in relation to the pre moment; ^‡^ Significant difference of the control session in relation to the pre moment.

### Secondary outcomes

The heart rate responses before and 30 min after the interventions are shown in figure 2E. The heart rate decreased after the control session compared to the pre-intervention period (74 [66-79] bpm and post 66 [60-72] bpm; p<0.001, Figure 2F).

**Figure 3:**
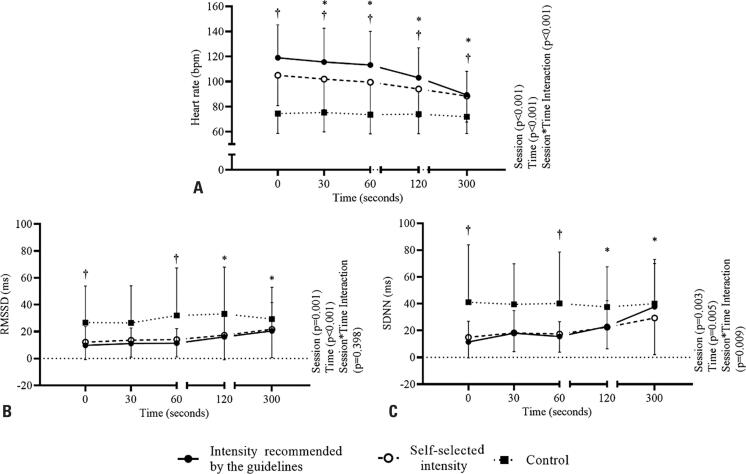
Autonomic modulation parameter responses during recovery after intervention

Figure 3 shows the heart rate recovery parameters obtained after each session. The heart rate (Figure 3A) after both exercise sessions was higher than that after the control session (p<0.001). In both exercise sessions, the heart rate decreased progressively over the 300 s recorded period (p<0.001 for all). The RMSSD (Figure 3B) increased significantly after both exercise sessions until 60 seconds after the intervention (p<0.05). The RMSSD values during these periods were lower in the exercise sessions than in the control session (p=0.014). The SDNN (Figure 3C) increased significantly after both exercise sessions until 120 seconds after the intervention (p<0.05). The SDNN values at 0 and 60 s were lower during the exercise sessions than during the control session (p=0.006 and p=0.046, respectively).

* Difference in exercise sessions compared to time 0; ^†^ Significant difference in exercise sessions in relation to the control session.

SDNN: standard deviation of all normal RR intervals recorded over a period; RMSSD: square root of the mean of the squared differences between adjacent normal RR intervals.

### Safety analysis

No adverse events were reported during any of the experimental sessions. The protocol was well-tolerated by all participants, including those with higher cardiovascular risk factors.

## DISCUSSION

The main findings of the current study were as follows: i) systolic blood pressure decreased similarly after both exercise sessions; ii) heart rate recovery involved a similar progressive decrease over time in both exercise sessions; and iii) after 30 min, heart rate decreased after the control session, whereas it increased after exercise sessions.

The results of this study demonstrated that both exercise interventions led to a significant acute decrease in systolic blood pressure, with a reduction of approximately 7mmHg. This reduction is a clinically relevant phenomenon in hypertensive populations^(25)^ indicating the positive impact of both sessions on blood pressure management. In the self-select intensity session, most patients (73.6%) opted to perform each set for a duration ranging from 5 to 10 minutes, while 26.4% preferred a duration exceeding 10 minutes^(10)^ The intensity levels ranged from 1.7km/h to 4.4km/h, with most patients (80.0%) exercising at speeds below 3.4 km/h, which is lower than the speed achieved during the intensity recommended by the guidelines session.^(10)^ As previously described,^(10)^ perceived exertion was similar between the conditions (5.7±1.7 vs. 5.1±1.8, p=0.167). Therefore, patients achieved longer exercise durations during the self-selected intensity session, completed the 30-minute exercise more quickly, and concluded the session earlier. The net effect of the intensity recommended by the guidelines session showed reductions of −8.1±13.2mmHg and −2.5±4.5mmHg in systolic and diastolic blood pressure, respectively. Conversely, the self-selected intensity session had a net effect of −8.6±10.3mmHg and −1.7±10.2mmHg for systolic and diastolic blood pressure, respectively. These results align with a prior study that observed significant blood pressure reductions after performing 15 bouts of 2-minute walking exercise, maintaining the heart rate at the onset of claudication pain in patients with PAD (*i.e*., −13.2±2mmHg and −5±2mmHg).^(6)^ The reductions in blood pressure observed under both conditions may be partially explained by autonomic adjustments, including parasympathetic reactivation, sympathetic withdrawal, and potential vascular adaptations. However, the absence of direct vascular assessments, such as endothelial function and arterial stiffness assessments, limits a more detailed exploration of these mechanisms. Future studies should incorporate these measures to enhance our understanding of the physiological responses to different exercise prescriptions in patients with PAD. These findings suggest that blood pressure reduction following walking in patients with PAD can be achieved through various prescriptions, offering different strategies for clinically relevant reductions, particularly among individuals with lower baseline systolic blood pressure values, as observed in the present study.

Heart rate decreased progressively after both exercise sessions, demonstrating a similar response to the return of resting cardiovascular function post-exercise. Previous studies have observed high reliability and good agreement in heart rate recovery assessments after the maximal treadmill exercise test in patients with PAD.^(26)^ Initially, the decrease in heart rate recovery was because of cardiac parasympathetic autonomic reactivation, followed by the withdrawal of cardiac sympathetic autonomic activity in the subsequent minutes.^(27)^ In this context, the RMSSD, a marker of cardiac parasympathetic modulation, after being reduced to the first 60 seconds after both exercise sessions compared to the control session, presented values similar to those of the control session after 120 seconds. This response pattern aligns with the observed interaction for heart rate, where post-exercise dynamics differed between sessions, but individual variability may have influenced the ability of the delta values to detect significant differences. These findings are interesting because heart rate recovery has been associated with a worse cardiovascular prognosis.^(27)^

The results of the current study have practical relevance, as they demonstrate the effectiveness of prescribing self-selected exercises in reducing systolic blood pressure and promoting heart rate recovery, similar to that achieved with exercises performed at the intensity recommended by the guidelines. In this context, many patients with PAD have an increased cardiovascular risk, including the presence of hypertension, among other comorbidities.^(28)^ Furthermore, prescribing self-selected exercises may be a strategy to increase long-term adherence to physical exercise in these individuals. Therefore, further research is needed to investigate whether chronic self-selected prescriptions are an effective and safe approach for improving blood pressure and other cardiovascular markers in patients with peripheral arterial disease.

This study had some limitations. First, we only analyzed a single session of exercise using a self-selected intensity, and the possible adherence and chronic effects of this prescription should be investigated further. Second, the study population was limited to patients with Stage II PAD. This intervention was used to examine the effectiveness of the exercise stimuli in patients with mild claudication (no tissue loss or gangrene), therefore limiting the generalizability of our findings to individuals with different disease severities.

## CONCLUSION

Acute walking exercise sessions performed at a self-selected intensity promoted a similar decrease in systolic blood pressure and heart rate recovery as walking exercise sessions performed at the intensity recommended by the guidelines.

## SUPPLEMENTARY MATERIAL Post-exercise effects of self-selected exercise intensity on cardiovascular parameters in peripheral artery disease: a randomized crossover study

Max Duarte de Oliveira, Hélcio Kanegusuku, Deivide Rafael Gomes de Faria, Tiago de Oliveira Peçanha, Nelson Wolosker, Marilia de Almeida Correia, Raphael Mendes Ritti-Dias

**DOI: 10.31744/einstein_journal/2025AO1498**

### Table 1S Response of blood pressure and heart rate between sessions after intervention in patients with peripheral arterial disease

**Table:**

	Intensity recommended by the guidelines		Self-selected intensity		Control		Time	Session	Interation
	Pre	Post	Pre	Post	Pre	Post			
Systolic blood Pressure, mmHg	119 114; 125	113 108; 119	122 116; 127	116*† 110; 121	122 117; 127	125 119; 130	0.018	0.001	0.001
Diastolic blood pressure, mmHg	70 66; 73	70 66; 73	69 64; 73	69 65; 73	70 67; 74	72 69; 76	0.374	0.208	0.057
Heart rate, bpm*	72 64; 80	75 68; 88	72 66; 78	71* 65; 76	73 65; 79	66† 59; 71	0.197	0.089	<0.001

* dif <0.05 *versus* Control;

† Dif <0.05 *versus* Pre. Data presented in mean and 95% confidence interval.

### Table 2S Delta response of blood pressure and heart rate between sessions after intervention in patients with peripheral arterial disease

**Table:**

	Intensity recommended by the guidelines	Self-selected intensity	Control	p value
	Δ (95%CI)	Δ (95%CI)	Δ (95%CI)	
Systolic blood Pressure, mmHg	-6.3* (-10.3; −2.3)	-6.2* (-10.2; −2.2)	2.5 (-1.7; 6.3)	0.003
Diastolic blood pressure, mmHg	-0.2 (-2.8; 2.4)	0.2 (-2.4; 2.8)	2.0 (-0.5; 4.7)	0.451
Heart rate, bpm	3.1 (-3.3; 9.5)	-1.4 (-7.8; 5.0)	0.3 (-6.1; 6.7)	0.619

* dif <0.05 *versus* control. Data presented in mean and 95% confidence interval.

### Table 3S Response of blood pressure and heart rate between sessions after intervention in patients with peripheral arterial disease (n=19)

**Table:**

	Time recovery (seconds)					Time	Session	Interation
	0	30	60	120	300			
Heart rate. bpm						<0.001	<0.001	<0.001
IRG	118 (107; 130)*	115 (104; 127)*†‡	113 (101; 124)*†‡	103 (92; 113)*†‡	89 (81; 97)*†‡			
SSI	104 (94; 115)*	101 (91; 112)*†‡	99 (89; 109)*†	93 (84; 103)*†‡	88 (79; 97)*†‡			
C	74 (67; 81)	75 (68; 82)	73 (67; 80)	74 (67; 80)	71 (66; 77)			
RMSSD. ms						0.001	0.001	0.398
IRG	9.8 (3.8; 15.8)*	11.1 (6.2; 16.0)	11.4 (6.8; 16.1)*	16.1 (9.0; 23.1)†	20.4 (11.4; 29.5)†			
SSI	12.1 (6.6; 17.7)*	13.6 (8.3; 19.0)	14.1 (8.6; 19.6)*	17.3 (9.5; 25.1)†	21.7 (12.7; 30.8)†			
C	26.8 (15.1; 38.4)	26.5 (14.7; 38.3)	32.0 (16.9; 47.2)	33.2 (18.3; 48.0)	29.3 (19.2; 39.5)			
SDNN. ms						0.003	0.005	0.009
IRG	11.5 (4.9; 18.1)*	18.0 (10.8; 25.2)	15.5 (10.8; 20.2)*	23.1 (14.9; 31.3)†	37.8 (22.7; 52.9)†			
SSI	14.9 (8.4; 21.5)*	18.2 (12.3; 24.2)	17.4 (11.6; 23.2)*	22.5 (15.6; 29.4)†	29.3 (17.7; 41.0)†			
C	41.1 (22.9; 59.4)	39.6 (26.7; 52.5)	40.2 (23.9; 56.6)	37.6 (24.8; 50.4)	40.0 (27.2; 52.8)			

* Dif. <0.05 *versus* Control;

† Dif. <0.05 *versus* Time recovery 0;

‡ Dif. <0.05 *versus* previous time.

IRG: intensity recommended by the guidelines; SSI: self-selected intensity; C: control.
